# Supplementary material for: Plant Aquaporin Gating Is Reversed by Phosphorylation on Intracellular Loop D—Evidence from Molecular Dynamics Simulations
Source: Int J Mol Sci. 2023 Sep 7;24(18):13798. doi: 10.3390/ijms241813798 (PMC10531447; doi:10.3390/ijms241813798)
Supplement: Supplementary file 1 [file ijms-24-13798-s001.zip › ijms-2562155-Supplementary Materials.pdf]

International Journal of
Molecular Sciences

MDPI

Supplementary Materials

Video S1. Opening of chain D. Schematic representation of *So*PIP2;1 tetrameric assembly extracted from experimental setup 1 simulation from time tstart = 500 nanoseconds to time tend = 600 nanoseconds. Each monomer is colored differently : in orange, chain A; in purple, chain B; in gray, chain C and in blue, chain D. Side chains of residues phosphorylated serine 188, arginine 187 and arginine 190 are represented. For chain D (in blue), we can see the establishment of salt bridges between phosphorylated serine 188 and arginine 187 first and then between phosphorylated serine 188 and arginine 190.
